# Exposure to airborne particulate matter during pregnancy is associated with preterm birth: a population-based cohort study

**DOI:** 10.1186/s12940-016-0094-3

**Published:** 2016-01-15

**Authors:** Emily DeFranco, William Moravec, Fan Xu, Eric Hall, Monir Hossain, Erin N. Haynes, Louis Muglia, Aimin Chen

**Affiliations:** Perinatal Institute, Cincinnati Children’s Hospital Medical Center, Cincinnati, OH USA; Maternal-Fetal Medicine, Department of Obstetrics and Gynecology, University of Cincinnati College of Medicine, 231 Albert Sabin Way, MSB Room 4553B, Cincinnati, OH 45267-0526 USA; Department of Environmental Health, University of Cincinnati College of Medicine, Cincinnati, OH USA; Division of Biostatistics and Epidemiology, Cincinnati Children’s Hospital Medical Center, Cincinnati, OH USA

**Keywords:** Air pollution, Particulate matter, PTB, Preterm birth, Prematurity

## Abstract

**Background:**

Test the hypothesis that exposure to fine particulate matter in the air (PM_2.5_) is associated with increased risk of preterm birth (PTB).

**Methods:**

Geo-spatial population-based cohort study using live birth records from Ohio (2007–2010) linked to average daily measures of PM_2.5_, recorded by 57 EPA network monitoring stations across the state. Geographic coordinates of the home residence for births were linked to the nearest monitoring station using ArcGIS. Association between PTB and high PM_2.5_ levels (above the EPA annual standard of 15 μg/m^3^) was estimated using GEE, with adjustment for age, race, education, parity, insurance, tobacco, birth season and year, and infant gender. An exchangeable correlation matrix for the monitor stations was used in the models. Analyses were limited to non-anomalous singleton births at 20-42weeks with no known chromosome abnormality occurring within 10 km of a monitor station.

**Results:**

The frequency of PTB was 8.5 % in the study cohort of 224,921 singleton live births. High PM_2.5_ exposure (>EPA recommended maximum) occurred frequently during the study period, with 24,662 women (11 %) having high exposure in all three trimesters. Pregnancies with high PM_2.5_ exposure through pregnancy had increased PTB risk even after adjustment for coexisting risk factors, adjOR 1.19 (95 % CI 1.09–1.30). Assessed per trimester, high 3^rd^ trimester PM_2.5_ exposure resulted in the highest PTB risk, adjOR 1.28 (95 % CI 1.20–1.37).

**Conclusions:**

Exposure to high levels of particulate air pollution, PM_2.5_, in pregnancy is associated with a 19 % increased risk of PTB; with greatest risk with high 3^rd^ trimester exposure. Although the risk increase associated with high PM_2.5_ levels is modest, the potential impact on overall PTB rates is robust as all pregnant women are potentially at risk. This exposure may in part contribute to the higher preterm birth rates in Ohio compared to other states in the US, especially in urban areas.

## Background

Since the industrial revolution, it has become increasingly evident that environmental toxicants contribute to human disease. Air pollution is associated with several acute and chronic cardiopulmonary diseases. Particulate air pollution, in particular, has been found to be harmful in numerous studies, and was 9^th^ leading risk factor in the 2010 Global Burden of Disease Study [1]. Particulate air pollution is a heterogeneous group of airborne matter that ranges in size from a few hundredths of a micrometer to visible particles up to 100 μm. Combustion is the main source of harmful particulate matter (PM). Fine particulate matter (PM_2.5_, referring to the upper limit of this fraction being 2.5 μm) has received much research and regulatory attention. As opposed to ultrafine particles – which are stable for only a short period of time, and coarse particles – whose travel is generally limited by the large size of the particles, PM_2.5_ can be both stable for long periods of time and are small enough to be distributed far from their source. PM_2.5_ comprises particles composed of hydrocarbons, organic compounds, ultrafine particle aggregates, biologic endotoxins, metals, and ions. Short-term exposure to PM_2.5_ can cause premature death, especially from cardiac and pulmonary disease. Long-term exposure to PM_2.5_ can also cause premature death from cardiac and pulmonary disease, but can also reduce lung development and lead to chronic respiratory diseases in children [2]. The EPA does monitor and set regulations for safe PM_2.5_ levels (www.epa.gov/ttn/naaqs/).

The connections between maternal toxin exposures and adverse birth outcomes is an emerging field of study that has begun to show that environmental toxicants are likely to be associated with some poor birth outcomes including stillbirth, low birthweight, some congenital anomalies, and preterm birth [3].

Several prior studies have reported the association between air pollutants and preterm birth, but report inconsistent findings of the association between preterm birth and increased PM_2.5_ levels [4–13]. Previous studies have been limited in design by exposures with measures at a single time point or with a lack of thorough adjustment for important clinical or socio-demographic risk factors. In this study we aim to integrate air quality measures from statewide monitoring stations with vital records to perform geospatial analyses testing the hypothesis that exposure to fine particles in the air (PM_2.5_) is associated with preterm birth risk.

## Methods

The Ohio Department of Health and Human Subjects Institutional Review Board approved a protocol for this study. This study was exempt from review by the Institutional Review Board at the University of Cincinnati, Cincinnati, Ohio. A data set generated from vital records of all live births that occurred in the state from 2007–2010 was provided for this analysis by the Ohio Department of Health, *n* = 597,000.

This is a geo-spatial population-based cohort study. The primary exposure was high level of airborne PM_2.5_, fine particulate matter in the air measuring <2.5 μm in diameter. Trimester-specific and total pregnancy average daily PM_2.5_ levels were categorized as high exposure if the average over the specified time period was greater than 15 μg/m^3^, which was the EPA annual standard during the period of study. A secondary analysis was then performed modeling PM_2.5_ levels as a continuous variable. The primary outcome was preterm birth was defined as delivery prior to 37 completed weeks of gestation. Gestational age was defined by the best obstetric estimate variable in the birth record, which combines last menstrual period and ultrasound parameters, as is commonly accepted in clinical practice for gestational age estimation. Analyses were limited to non-anomalous singleton live births.

**Fig. 1:**
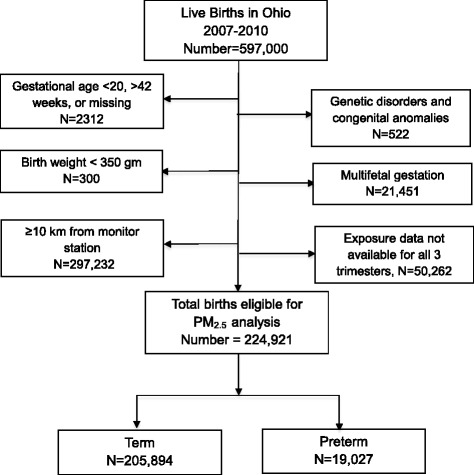
Flow diagram of the study population, Ohio births 2007–2010

Daily measures of PM_2.5_, recorded by 57 monitoring stations across the state of Ohio, were obtained from the Environmental Protection Agency from 2007–2010 and monthly averages were calculated for each station [14]. Maternal address at time of birth was geocoded and distance to the nearest EPA monitoring station was calculated using ArcGIS 10.1 (ESRI, Redlands, CA) software. Births at gestational ages 20 to 42 weeks with a maternal home address within 10 km of an EPA PM_2.5_ monitoring station were included in this analysis (*n* = 224,921, Fig. 1). Average monthly PM_2.5_ levels from the nearest EPA station were linked to the birth record data. Average PM_2.5_ levels for each trimester and an average for the entire pregnancy were calculated for each birth included in this analysis.

Demographic, medical and delivery characteristics of preterm live births (<37 weeks) were compared to term live births (37–42 weeks) using *t*-test for continuous variable comparisons and *χ*^2^ tests for categorical variables. The association between preterm birth and high PM_2.5_ exposure was estimated using generalized estimating equations (GEE), with adjustment for the confounding influences of age, race, education, parity, insurance, tobacco, birth season and year, and infant sex. Odds ratios for high PM_2.5_ exposure during the first, second, and third trimester as well as high exposure averaged over the entire pregnancy were estimated from separate models with adjustment for all factors listed above. Covariates include in the models were chosen based on significant differences noted among bivariate comparisons, biologic plausibility, and parsimony within the model.

An exchangeable correlation matrix for the monitoring stations was used in the GEE models to account for spatial correlation within the same PM_2.5_ monitor. Analyses were performed using SAS version 9.3, SAS Institute Inc., Cary, NC, USA. Comparisons with a probability value <0.05 or 95 % confidence interval without inclusion of the null were considered statistically significant. Population attributable risk percentage (PAR%) was calculated as: PAR% = 100 × Pe (RR - 1)/(Pe(RR-1) + 1), where Pe is percentage of high exposure in the entire population (approximately 10 % for the population included in this study) and RR is relative risk. Odds ratios approximate the relative risk in studies with an outcome rate of less than 10 %, as in this study. Therefore we utilized estimated odds ratios in lieu of relative risk in the PAR calculation. The PAR calculation inherently assumes a causal relationship, which is yet unproven in the association of particulate matter and preterm birth. With this in mind, we provide this calculation in effort to estimate the potential contribution of high PM_2.5_ exposure to preterm birth on a population level.

## Results

The study population included 224,921 singleton non-anomalous live births: 19,027 preterm births and 205,894 term births, Fig. 1. The preterm rate decreased during the study period, from 8.6 % in 2007 to 8.2 % in 2010, Table 1. Most births analyzed (97 %) occurred in very urban areas, where most monitoring stations are located and exposure levels are likely to be highest. Preterm birth rates were higher among the oldest mothers, age ≥40 years, 10.9 %, and non-Hispanic black mothers, 11.2 %, as well as women with lower education level and tobacco use. Women with no prenatal care had the highest rate of singleton preterm birth, 19.8 %. Season of conception had no influence on preterm birth rate.

**Table 1:** Maternal Characteristics, Ohio 2007–2010

	Preterm (%) *N* = 19,027	Term (%) *N* = 205,894	*p*-value	Total births (%) *N* = 224,921
Demographic factors				
Advanced maternal age				
35 – 39 years	1901 (10.0)	19,302 (9.4)	<0.01	21,203 (9.4)
≥40 years	482 (2.5)	3952 (1.9)		4434 (2.0)
Race and ethnicity				
Non-Hispanic White	9940 (52.7)	127,636 (62.6)	<0.01	137,576 (61.2)
Non-Hispanic Black	7507 (39.8)	59,630 (29.2)		67,137 (30.1)
Hispanic	937 (5.0)	10,583 (5.2)		11,520 (5.2)
Other	457 (2.4)	6.116 (3.0)		6573 (2.9)
Social behaviors & socioeconomic factors				
Education				
Less than high school	4778 (25.4)	40,658 (19.9)	<0.01	45,436 (20.4)
High school graduate	5127 (27.3)	50,721 (24.9)		55,848 (25.1)
Some postsecondary	8875 (47.3)	112,590 (55.2)		121,465 (54.5)
Tobacco use	4568 (24.0)	38,292 (18.6)	<0.01	42,860 (19.1)
Medicaid insurance	9672 (50.8)	87,631 (42.6)	<0.01	97,303 (43.3)
Prenatal care initiation				
First trimester	8051 (61.6)	103,232 (67.7)	<0.01	111,283 (67.2)
Second trimester	3219 (24.6)	36,882 (24.2)		40,101 (24.2)
Third trimester	708 (5.4)	7897 (5.2)		8605 (5.2)
No prenatal care	1092 (8.4)	4422 (2.9)		5514 (3.3)
Year of birth				
2007	5234 (27.5)	55,325 (26.9)	0.01	60,559 (26.9)
2008	4969 (26.1)	52,708 (25.6)		57,677 (25.6)
2009	4600 (24.2)	50,364 (24.5)		54,964 (24.4)
2010	4225 (22.2)	47,497 (23.1)		51,721 (23.0)
Season				
Winter	4755 (25.0)	50,205 (24.4)	0.26	54,960 (24.4)
Spring	4787 (25.1)	51,827 (25.2)		56,614 (25.2)
Summer	4835 (25.4)	53,173 (25.8)		58,008 (25.8)
Fall	4650 (24.4)	50,689 (24.6)		55,339 (24.6)

Dichotomous variables for first 2 columns are presented as percent of total for each characteristic

Continuous variables are presented as median (IQR) for non-normally distributed data and mean +/- standard deviation for normally distributed data

**Fig. 2:**
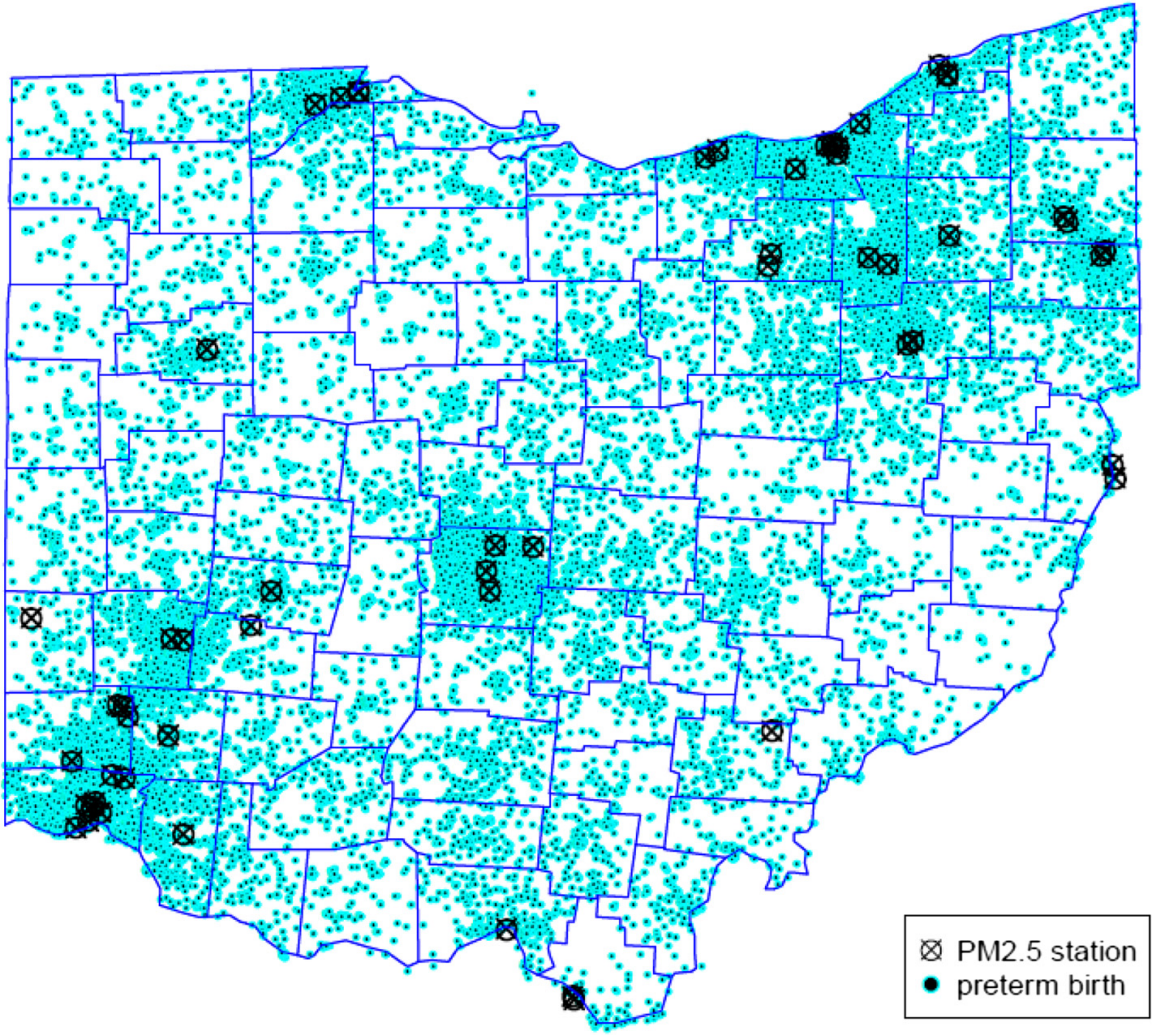
Preterm births and PM_2.5_ monitoring stations in Ohio, 2007–2010

The locations of PM_2.5_ monitor stations in Ohio and preterm births during the study period are demonstrated in Fig. 2. The mean PM_2.5_ level during the study period (2007–2010) in Ohio was 13.03 μg/m^3^ [±1.57 μg/m^3^, IQR (Q1: 11.84, Q3: 14.13, IQR: 2.3)], which is higher than the current US EPA National Ambient Air Quality Standard (NAAQS) of 12 μg/m^3^. Mean PM_2.5_ level, IQR for the entire cohort per trimester was: 1^st^ trimester 13.19, 2.36 μg/m^3^; 2^nd^ trimester 12.98, 2.08 μg/m^3^; 3^rd^ trimester 12.93, 2.30 μg/m^3^.

The frequency of high exposure to PM_2.5_ greater than the EPA standard of 15 μg/m^3^ ranged from 17 to 20 % for each trimester of pregnancy, and 11 % of parturients had high exposure throughout pregnancy in all three trimesters. The frequency of high PM_2.5_ exposure was higher in preterm births compared to term births, see Table 2. The preterm birth rate was also increased with high PM_2.5_ exposure during the first and third trimester, and when there was high PM_2.5_ exposure averaged over the entire pregnancy, as demonstrated in Table 3. Likewise, the mean PM_2.5_ levels during each trimester when compared between term and preterm pregnancies demonstrated significantly higher average PM_2.5_ levels during the first and third trimester for the preterm birth group compared to term births, *p* = 0.01 (data not displayed in table). Likewise, the overall pregnancy average PM_2.5_ levels were higher for the preterm birth group compared to the term birth group, *p* < 0.01.

**Table 2:** PM_2.5_ levels in Ohio 2007 – 2010, by trimester of exposure in pregnancy and preterm status

	Preterm births *N* = 19,027	Term births *N* = 205,894		All live births *N* = 224,921
	% PM_2.5_ ≥ 15 μg/m^3^	% PM_2.5_ ≥ 15 μg/m^3^	*p*-value	% PM_2.5_ ≥ 15 μg/m^3^
First trimester	22.97	21.81	<0.001	21.91
Second trimester	17.30	17.36	0.835	17.36
Third trimester	22.84	18.90	<0.001	19.23
Entire pregnancy	12.94	10.78	<0.001	10.96

% PM_2.5_ ≥ 15 μg/m^3^ = percent of births in Ohio with average exposure level exceeding the EPA standard of 15 μg/m^3^

**Table 3:** Preterm birth rate by PM_2.5_ levels in Ohio 2007 – 2010 and trimester of exposure in pregnancy

	PM_2.5_ < 15 μg/m^3^		PM_2.5_ ≥ 15 μg/m^3^		
	n	% Preterm	n	% Preterm	*p*-value
First trimester	175,649	8.34	49,272	8.87	<0.001
Second trimester	185,883	8.47	39,038	8.43	0.835
Third trimester	181,665	8.08	43,256	10.05	<0.001
Entire pregnancy	200,259	8.27	24,662	9.99	<0.001

% preterm represents the rate of birth <37 weeks of gestational age among the study cohort of singleton non-anomalous live births

**Fig. 3:**
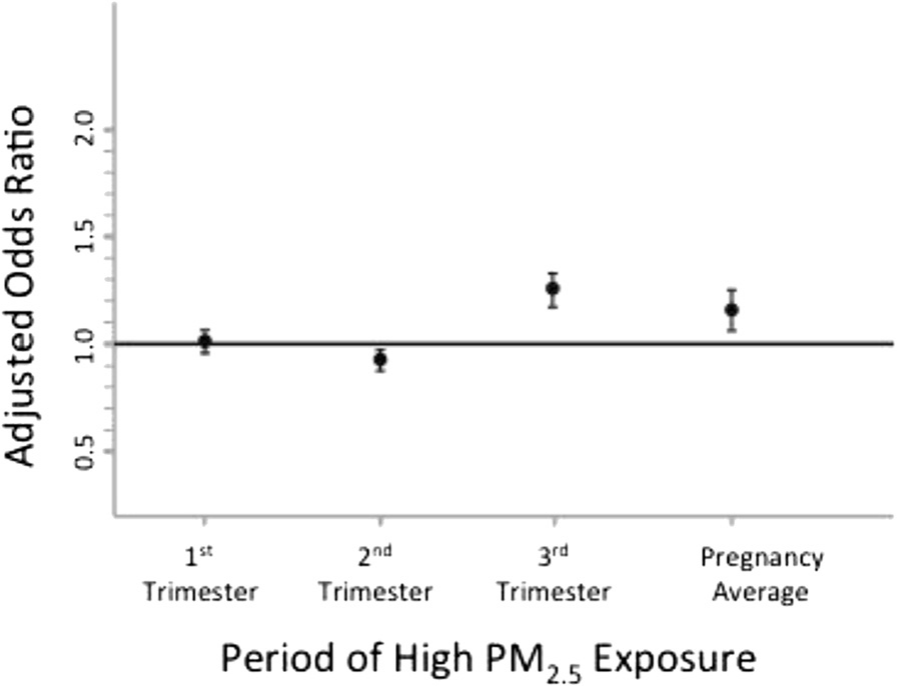
Relative risk of preterm birth associated with exposure to high levels of PM_2.5_, by trimester of pregnancy, Ohio 2007–2010

Logistic regression models were constructed to identify factors associated with preterm birth. Factors with significant associations included maternal age > 35, non-Hispanic black race, Hispanic ethnicity, high school education or less, no prenatal care, tobacco use, high PM_2.5_ exposure during the third trimester, and high PM_2.5_ exposure over the entire pregnancy (Table 4 and Fig. 3). High PM_2.5_ levels > 15 μg/m^3^ during the third trimester or high when averaged over the entire pregnancy were associated with an increased risk of preterm birth <37 weeks of gestation, adjOR 1.28 (CI 1.20, 1.37) and adjOR 1.19 (CI 1.09, 1.30), respectively (Table 4). When PM_2.5_ exposure was modeled as a continuous variable in a secondary analysis, no significant association between exposure and preterm birth was observed: adjOR 0.99 (CI 0.98, 1.00) for first trimester, adjOR 0.98 (CI 0.97, 1.00) second trimester, adjOR 1.0 (CI 0.99, 1.00) third trimester, and pregnancy average adjOR 0.98 (CI 0.96, 1.0).

**Table 4:** Logistic regression of factors associated with preterm birth, Ohio 2007-2010

	Adjusted odds ratio^2^	95 % confidence interval
Maternal age, years		
<20	0.93	0.84, 1.02
20–24	0.99	0.94, 1.05
25–29	1.00	Referent
30–34	1.04	0.97, 1.10
35–39	1.27	1.19, 1.36
≥40	1.52	1.37, 1.69
Maternal race		
Non-Hispanic white	1.00	Referent
Non-Hispanic black	1.46	1.36, 1.57
Hispanic	1.10	1.01, 1.18
Other Non-Hispanic	1.02	0.88, 1.18
Maternal education level		
Less than high school	1.13	1.07, 1.19
High school only	1.23	1.17, 1.29
Postsecondary education	1.00	Referent
Prenatal care initiation		
First trimester	1.00	Referent
Second trimester	0.97	0.94, 1.01
Third trimester	0.94	0.85, 1.05
No prenatal care	2.51	2.22, 2.84
Tobacco Use	1.28	1.22, 1.35
High PM_2.5_ exposure^2^		
Average over pregnancy	1.19	1.09, 1.30
First trimester	1.02	0.97, 1.07
Second trimester	0.96	0.90, 1.01
Third trimester	1.28	1.20, 1.37

1. Odds ratio estimates for covariates are adjusted for other factors listed in the first column of the table as well as parity, infant sex, year of birth, season of birth, and insurance type in the model with high average PM_2.5_ exposure over pregnancy

2. The odds ratio estimates for first, second, third trimester high Pm2.5 exposure are from separate models with adjustment for the same covariates as listed above

The attributable risk of preterm birth related to high PM_2.5_ exposure > 15 μg/m^3^ was 0.0172 (95 % CI 0.0135 – 0.0208). The attributable risk percent: (Ie-Iu)/Ie is 17.18 (13.49 – 20.86) %, i.e. for the exposed population, 17.18 % of preterm births were from high PM_2.5_ exposure. In our study sample in which 10.96 % of the population was exposed to high PM_2.5_, the population attributable risk percentage of high PM_2.5_ exposure was 2.22 % for preterm birth.

## Discussion

Our study adds to the growing body of evidence that particulate matter in the air is deleterious to human health. In our study sample, exposure to high levels of PM_2.5_ > 15 μg/m^3^ was significantly associated with preterm birth – both when high exposure occurred on average over the course of pregnancy and with high exposure limited to just the third trimester. Our calculated attributable risk percent estimates that decreasing PM_2.5_ levels below this EPA standard threshold could theoretically decrease preterm birth by 17.18 % in the exposed group, corresponding to a 2.22 % decrease in preterm birth rate in the population.

An association between air pollution and preterm birth was first suggested in an analysis of the Nashville Air Pollution Study that showed increased risk of death in preterm infants whose mothers were likely to be exposed with high levels of particulate pollution [15]. Subsequent studies have further explored the link between preterm birth and other air pollutants using measurements of a variety of pollutants (TSP, SO2, NOx, CO, and PM_10_) in proximity to maternal residence, measured by stationary or remote sensing satellite monitors. These studies demonstrated mixed results with regard to associations with preterm birth and timing of high exposure periods during the pregnancy [16–21].

Prior studies that have attempted to link preterm birth specifically to PM_2.5_ have also reported varied results. Gray et al. did not find significant effects in a study using EPA modeled data in North Carolina [6]. In a multi-country study, Fleisher found significant differences in preterm birth only in China when comparing areas with very high average PM_2.5_ levels (>36.5 μg/m^3^) compared to low levels (<12.5 μg/m^3^) [22]. Recent studies using satellite measurements have also been mixed: Rudra [23] found no effect, Gehring [5] found a non-significant trend toward increased risk of preterm birth with increased PM_2.5_ exposure, while Lee (2013) found high first trimester exposure was associated with modestly increased risk (OR 1.10 [1.01–1.20]) [24]. Jalaludin found that high PM_2.5_ levels associated only with risk of preterm birth in pregnancies conceived in the winter (OR 1.426) [25]. Kloog found an increased risk of prematurity using a model that incorporated measured and estimated exposure values (OR 1.06 (1.01–1.13) per 10 μg/m^3^ increase in third trimester) [9]. A recent meta-analysis combining previous studies found a modestly increased risk of preterm birth for each 10 μg/m^3^ increase in PM_2.5_ (OR 1.10 [1.03–1.18]); however, they did not find evidence of increased risk in trimester specific exposures, as we identified in this study [26].

The PM_2.5_ concentration threshold at which health is harmed is not well defined. We used the cutoff of 15 μg/m^3^ average to define high exposure as this was the EPA National Ambient Air Quality standard for annual mean levels of PM_2.5_ during the time period of the study. The standard was reduced to 12 μg/m^3^ in 2012, after the EPA determined that the threshold level for harm may be in the 13–14 μg/m^3^ range [27]. We used the cutoff of 15 μg/m^3^ in these analyses because it may be more representative of exposure levels in relation to the EPA standard in our region during the time period of study. Using this threshold to define high exposure, we identified significant associations with preterm birth risk. However, when PM_2.5_ exposure was modeled as a continuous variable, no risk increase for preterm birth was identified. This may suggest a threshold effect in which a critical level of exposure is necessary before harmful health effects are seen.

There are multiple mechanisms by which particulate matter may lead to deleterious health effects. Three broad pathways exist that may explain how air pollution may affect organs outside of the lung: 1) Toxic substances may enter the blood via the lungs and be carried throughout the circulation. 2) There may be a systemic oxidative stress and inflammatory response that is either triggered in the lungs, or by substances once they gain access to the general circulation. 3) Toxicants may disrupt the autonomic nervous system causing imbalance of sympathetic and parasympathetic systems. There is evidence of the importance for each of these pathways at the cellular and molecular level in non-pregnant humans, especially related to cardiovascular events [28]. It is plausible that any or all of these mechanisms may contribute to alterations in uteroplacental perfusion, nutrient and oxygen transfer to the fetus, or stimulate the inflammatory response that commonly precedes the onset of preterm parturition.

There are a number of methodological limitations of our study, which are common to studies examining exposure to ambient air pollution and birth outcomes: 1) Specific pollutants and their ambient concentrations not considered independently but rather grouped as PM_2.5_. 2) Individual level exposure not quantified by personal sampling. Quantification of an individual’s exposure is imprecise without either a completely controlled environment or portable sampling equipment – both of which are not practical for population cohort studies. 3) It is not known when the critical time for exposure is for specific outcomes. 4) Birth certificate data may not adequately describe potentially confounding socioeconomic or medical information. Regarding exposure quantification, there is likely some degree of sampling bias as EPA measurement stations are not randomly placed. Rather, monitoring sites are intended to capture ambient air levels and may be strategically placed to avoid major industrial sources of air, which could contribute to selection, and information bias. This misclassification could bias the results toward the null, and contribute to the lack of significant association identified when exposure was modeled in quartiles or as a continuous variable. Additionally, categorizing third trimester exposure as high levels occurring after 28 weeks would not account for early preterm births that occur prior to the third trimester. Despite statistical adjustment for available socioeconomic factors, unidentified socioeconomic factors or co-existent environmental risk factors may confound the interpretation of results in our study or similar studies. More populated areas tend to have higher proportions of patients with socioeconomic risk factors for preterm birth. Likely the best way to truly determine the connection between air pollution and preterm birth would be to have subjects carry continuous pollutant monitoring systems. This would allow more precise assessment of the threshold of risk and would allow us to further define behaviors associated with high pollutant exposure.

## Conclusions

Our data suggest that women exposed to higher than the EPA standard exposure level of PM_2.5_ over the course of pregnancy are at increased risk for preterm birth. Based on trimester-specific high exposure periods, high exposure during the third trimester of pregnancy are also significantly associated with increased preterm birth risk. While this study does not precisely define a safe threshold for exposure, it does support the EPA’s decision to decrease the standard exposure limit of PM_2.5_ concentration for individuals in the US. Additional research is needed to determine individual-level exposure as measured by ambient air and internal biomarkers of exposure and effect.

## Abbreviations

adjOR: adjusted odds ratio
CO: carbon monoxide
EPA: Environmental Protection Agency
GEE: generalized estimating equations
NAAQS: US EPA National Ambient Air Quality Standard
NOx: nitrogen oxide
OR: odds ratio
PAR: Population attributable risk
PM_10_: fine particulate matter, smaller than 2.5 μm
PM_2.5_: Fine particulate matter smaller than 2.5 μm
PTB: Preterm birth
RR: relative risk
SO2: sulfer dioxide
TSP: trisodium phosphate
